# Tuberculosis and osteoporotic fracture risk: development of individualized fracture risk estimation prediction model using a nationwide cohort study

**DOI:** 10.3389/fpubh.2024.1358010

**Published:** 2024-04-24

**Authors:** Hayoung Choi, Jungeun Shin, Jin-Hyung Jung, Kyungdo Han, Wonsuk Choi, Han Rim Lee, Jung Eun Yoo, Yohwan Yeo, Hyun Lee, Dong Wook Shin

**Affiliations:** ^1^Division of Pulmonary, Allergy, and Critical Care Medicine, Department of Internal Medicine, Hallym University Kangnam Sacred Heart Hospital, Seoul, Republic of Korea; ^2^International Healthcare Center, Samsung Medical Center, Seoul, Republic of Korea; ^3^Department of Biostatistics, College of Medicine, Catholic University of Korea, Seoul, Republic of Korea; ^4^Department of Statistics and Actuarial Science, Soongsil University, Seoul, Republic of Korea; ^5^Department of Internal Medicine, Chonnam National University Hwasun Hospital, Chonnam National University Medical School, Hwasun, Republic of Korea; ^6^Department of Family Medicine and Supportive Care Center, Samsung Medical Center, Sungkyunkwan University School of Medicine, Seoul, Republic of Korea; ^7^Department of Family Medicine, Healthcare System Gangnam Center, Seoul National University Hospital, Seoul, Republic of Korea; ^8^Department of Family Medicine, Hallym University Dongtan Sacred Heart Hospital, Hallym University College of Medicine, Hwaseong, Republic of Korea; ^9^Division of Pulmonary Medicine and Allergy, Department of Internal Medicine, Hanyang Medical Center, Hanyang University College of Medicine, Seoul, Republic of Korea; ^10^Department of Family Medicine, Supportive Care Center, Samsung Medical Center, Sungkyunkwan University School of Medicine, Seoul, Republic of Korea; ^11^Department of Clinical Research Design and Evaluation, Samsung Advanced Institute for Health Science and Technology (SAIHST), Sungkyunkwan University, Seoul, Republic of Korea

**Keywords:** tuberculosis, survivor, fractures, epidemiology, risk factor

## Abstract

**Purpose:**

Tuberculosis (TB) is linked to sustained inflammation even after treatment, and fracture risk is higher in TB survivors than in the general population. However, no individualized fracture risk prediction model exists for TB survivors. We aimed to estimate fracture risk, identify fracture-related factors, and develop an individualized risk prediction model for TB survivors.

**Methods:**

TB survivors (*n* = 44,453) between 2010 and 2017 and 1:1 age- and sex-matched controls were enrolled. One year after TB diagnosis, the participants were followed-up until the date of fracture, death, or end of the study period (December 2018). Cox proportional hazard regression analyses were performed to compare the fracture risk between TB survivors and controls and to identify fracture-related factors among TB survivors.

**Results:**

During median 3.4 (interquartile range, 1.6–5.3) follow-up years, the incident fracture rate was significantly higher in TB survivors than in the matched controls (19.3 vs. 14.6 per 1,000 person-years, *p* < 0.001). Even after adjusting for potential confounders, TB survivors had a higher risk for all fractures (adjusted hazard ratio 1.27 [95% confidence interval 1.20–1.34]), including hip (1.65 [1.39–1.96]) and vertebral (1.35 [1.25–1.46]) fractures, than matched controls. Fracture-related factors included pulmonary TB, female sex, older age, heavy alcohol consumption, reduced exercise, and a higher Charlson Comorbidity Index (*p* < 0.05). The individualized fracture risk model showed good discrimination (concordance statistic = 0.678).

**Conclusion:**

TB survivors have a higher fracture risk than matched controls. An individualized prediction model may help prevent fractures in TB survivors, especially in high-risk groups.

## Introduction

Tuberculosis (TB) is the most common communicable respiratory disease worldwide. It is a major public health issue in the Republic of Korea, with an estimated incidence of 31 cases per 100,000 people in 2023 (1, 2). Even after completing effective treatment, TB infections may affect TB survivors’ health by chronically elevating systemic inflammatory cytokines (3), leading to various complications other than respiratory diseases, such as ischemic heart disease (4) and dementia (5).

Osteoporotic fractures, including hip and vertebral fractures, are a major cause of morbidity and mortality in the aging population (6), with an increasing burden on the healthcare system (7). Studies have found that proinflammatory cytokines induce osteoclast bone resorption, consequently increasing the risk of osteoporotic fractures (8). Thus, chronic inflammatory disorders, such as chronic obstructive pulmonary disease (COPD), rheumatoid disease, and inflammatory bowel disease, are associated with osteoporosis and increased fracture risk (9).

While increased fracture risk is related to chronic inflammatory disease, data on the association between TB and the incidence of fractures are limited. A Taiwanese retrospective cohort study compared the incidence of osteoporosis and osteoporotic fractures between TB survivors and matched controls. After adjusting for variables including sociodemographic (income level and urbanization) and co-morbidities, TB survivors showed around two-fold higher risk of osteoporosis and osteoporotic fracture than matched controls (10). However, this study was limited in that important osteoporosis-related factors, including body mass index (BMI), smoking habits, and alcohol consumption, need to be adjusted for, as nutritional status affects osteoporosis incidence (11). A Korean study that further adjusted for BMI, smoking status, and alcohol consumption revealed an approximately 1.3-fold higher risk of osteoporosis and osteoporosis-related fractures in TB survivors than in matched controls (12); however, the study lacked a prediction model for fractures in TB survivors. The development of an individualized prediction model for estimating future fracture risk would be a cost-effective strategy for preventing fractures in TB survivors.

Hence, this study aimed to assess the association between a previous TB infection history and the risk of fractures while comprehensively adjusting for confounders, identifying fracture-related risk factors, and developing a fracture prediction model for TB survivors.

## Methods

### Data source and study setting

The National Health Insurance Service (NHIS) in the Republic of Korea is a mandatory, universal public health insurance system that covers approximately 97% of the Korean population and provides medical aid to 3% of the lowest-income population. All medical services supplied by healthcare providers are reimbursed by the NHIS.

The NHIS database includes (1) a qualification database containing information on age, sex, income, region, and eligibility type; (2) a claim database providing general information on specifications and statements on diagnosis defined by the International Classification of Disease 10th revision (ICD-10), consultation, and prescription; (3) a health screening database; and (4) death information (13–18). The health screening database encompasses data from annual or biennial health screening examination programs for all adults; details are provided in previous studies (13–18).

### Study population

**Figure 1:**
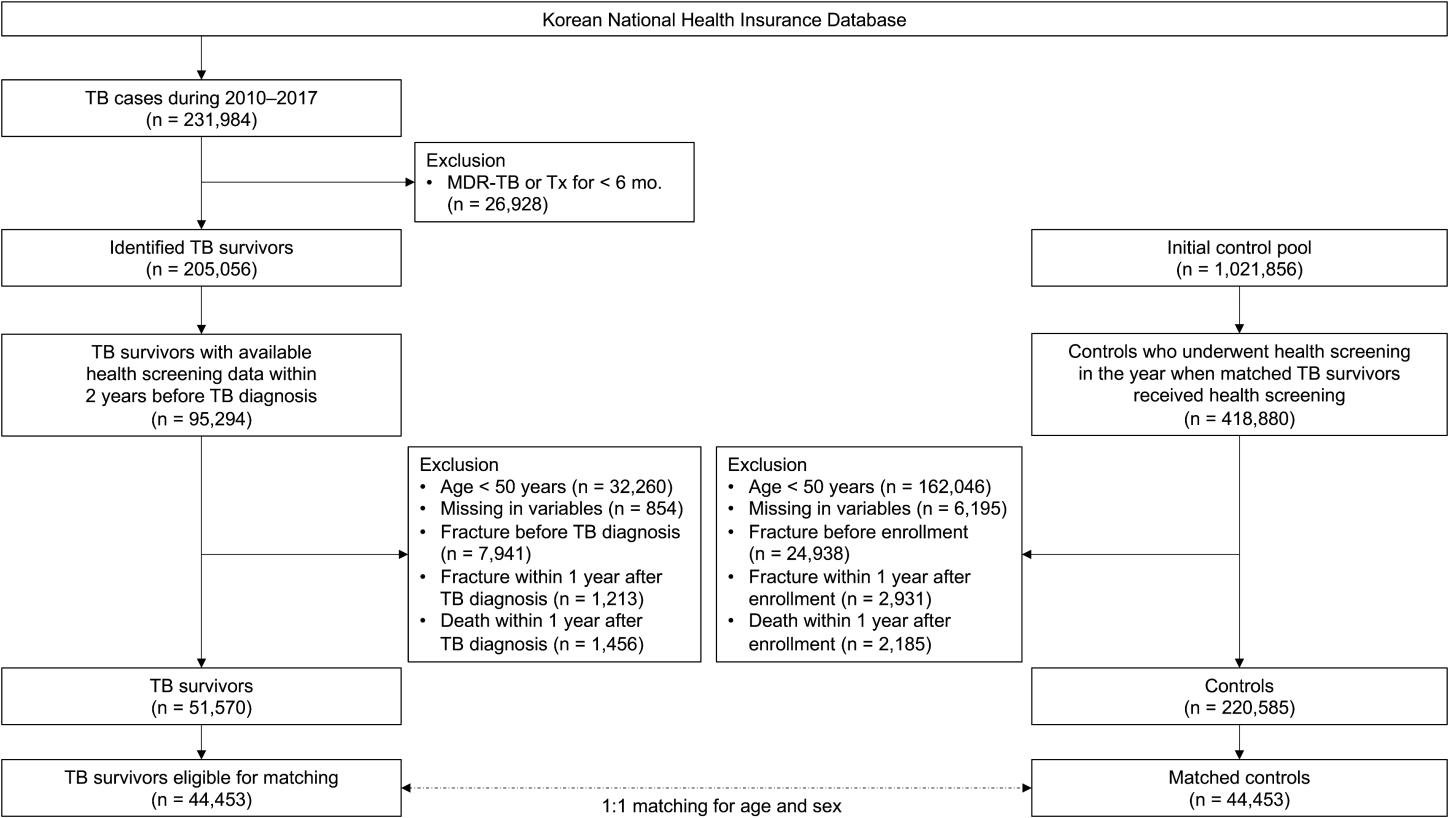
Flow chart of the study population. TB, tuberculosis; MDR, multidrug resistant.

To establish a TB survivor cohort, among the 231,984 patients diagnosed with TB between 2010 and 2017, 26,928 diagnosed with multidrug-resistant TB or treated for less than 156 days were excluded. Of the identified 205,056 TB survivors, this study initially included 95,294 who underwent health screening within 2 years before the date of TB diagnosis. After excluding 32,260 participants aged <50 years, 854 participants with missing information, 7,941 with a history of fracture before TB diagnosis, 1,213 with an occurrence of fracture within 1 year of TB diagnosis, and 1,456 who died within 1 year of TB diagnosis, 51,570 TB survivors remained. Of the 51,570 TB survivors, 44,453 were eligible for 1:1 age and sex matching (Figure 1).

To establish a control group, we performed 1:1 matching sequentially by year to match TB survivors diagnosed with TB in a specific year to eligible living control participants in the same year based on age (≥ 50 years) and sex. Ages of study participants were changed to integers, and then the ages of the TB survivors and controls were exactly matched. Control participants were allotted an index date identical to the date of TB diagnosis of matched TB survivors. Finally, 44,453 TB survivors and 44,453 matched controls were included in this study (Figure 1).

This study was approved by the Institutional Review Board of the Hanyang University Hospital (application no. HYUH-2019-10-047). The review board waived the requirement for written informed consent because the data were publicly available and anonymized under confidentiality guidelines.

### Study exposure, outcomes, and follow-up

The primary exposure in this study was TB, and the inclusion criteria were as follows: (1) patients with at least two healthcare uses, including outpatient department visits, emergency department visits, or hospitalizations, with ICD-10 codes for TB (A15-19) and the specific NHIS codes for TB (V206, V246, and V000); and (2) patients who were prescribed two or more of the following anti-TB drugs for at least 90 days. More details are provided in a previous study (4, 5, 13, 16, 19). TB was classified as pulmonary TB (A15, A16, or A19) or extrapulmonary TB (A17 or A18 without A15, A16, or A19).

The primary endpoint was the occurrence of new fractures during the follow-up period. Fracture incidence was defined using the ICD-10 code for each fracture type (7, 20–22). Vertebral fractures were defined by ICD-10 codes S220, S221, S320, S327, M484, and M485 and at least two related outpatient clinic visits (23), whereas hip fractures were defined by ICD-10 codes S720 and S721 and related hospitalizations (7). All other fractures were defined by ICD-10 codes S420, S422, S423, S525, S526, S823, S825, and S826, and more than two related visits to an outpatient clinic. This study followed the participants from 1 year after the index date to the date of the fracture event, death, or until the last follow-up date (December 31, 2018), whichever came first.

### Covariates

Socioeconomic position (location of residence and level of income), lifestyle factors (smoking, alcohol consumption, and physical activity), and BMI were considered potential covariates (24–32). Smoking status was categorized into three groups: never, former, and current smokers. Alcohol consumption was divided into non-, mild to moderate (< 30 g of alcohol/day), and heavy (≥ 30 g of alcohol/day) drinkers (33). Regular exercise was defined as ≥30 min of moderate physical activity ≥ five times per week or ≥ 20 min of vigorous physical activity ≥ three times per week (34, 35). BMI was calculated by dividing the weight (kg) by the height in meters squared (m^2^). Participants’ BMIs were divided into the following groups: underweight (<18.5 kg/m^2^), normal (18.5–22.9 kg/m^2^), overweight (23.0–24.9 kg/m^2^), and obese (≥ 25.0 kg/m^2^) following the recommendations for Asians by the World Health Organization (36). The overall comorbidity load was evaluated using the Charlson Comorbidity Index (CCI) (37, 38).

### Statistical analysis

The baseline characteristics of TB survivors were compared with those of matched controls using a two-tailed Student’s *t*-test for continuous variables and a χ^2^-test for categorical variables. The cumulative incidence of fractures was calculated by dividing the number of incident cases by the total follow-up duration (1,000 person-years). A cumulative incidence plot was used to compare the incidence of fractures between TB survivors and matched controls, and a log-rank test was used to evaluate significant differences between the two groups. Cox proportional hazards models were used to evaluate fracture risk in TB survivors and explore related factors. Model 1 was an unadjusted model, and Model 2 was adjusted for age, sex, BMI, smoking status, alcohol consumption, socioeconomic position (income level and residential area), and regular exercise.

To develop a fracture prediction model for TB survivors, we applied 10 variables (TB type, age, sex, BMI, smoking, alcohol consumption, regular exercise, income level, residential area, and CCI) as weighted risk scores based on the beta coefficients of each variable in the Cox proportional hazards model by assigning scores (0–100). The total score ranged from 0 to 270. Model selection was conducted, including all selected variables considered significant in the univariable analysis. Model discrimination was evaluated using the concordance statistic (c-statistic). SAS version 9.4 (SAS Institute Inc., Cary, NC, United States) was used for statistical analyses, and *p*-values <0.05 were considered statistically significant.

## Results

### Baseline characteristics

The mean age of the TB survivors was 64.5 years, and 60.8% were male. The proportions of participants in the lowest income quantile (19.8% vs. 18.6%) and ever smokers (43.4% vs. 40.2%) were higher in TB survivors than those in the matched controls, whereas the proportions of alcohol drinkers (36.8% vs. 38.5%) and regular exercisers (19.4% vs. 23.4%) were lower in TB survivors than those in the matched controls (*p* < 0.001 for all). Additionally, TB survivors showed significantly lower BMI (mean, 22.4 kg/m^2^ vs. 24.1 kg/m^2^) and a higher CCI (2.7 vs. 1.6) than the matched controls (*p* < 0.001 for both) (Table 1).

**Table 1:** Baseline characteristics of the study subjects.

Variable	Total (*n* = 88,906)	TB survivors (*n* = 44,453)	Matched controls (*n* = 44,453)	*p*-value
Age, years	64.5 ± 9.0	64.5 ± 9.0	64.5 ± 9.0	1.0
50–59	31,894 (35.9)	15,947 (35.9)	15,947 (35.9)	
60–69	28,264 (31.8)	14,132 (31.8)	14,132 (31.8)	
70–79	24,386 (27.4)	12,193 (27.4)	12,193 (27.4)	
≥80	4,362 (4.9)	2,181 (4.9)	2,181 (4.9)	
Sex				
Male	54,032 (60.8)	27,016 (60.8)	27,016 (60.8)	1.0
Female	34,874 (39.2)	17,437(39.2)	17,437(39.2)	
Residential area				0.968
Rural	38,440 (43.2)	19,223 (43.2)	19,217 (43.2)	
Urban	50,466 (56.8)	25,230 (56.8)	25,236 (56.8)	
Low income*	17,081(19.2)	8,808 (19.8)	8,273 (18.6)	<0.001
Smoking status				
Never	51,721 (58.2)	25,159 (56.6)	26,562 (59.8)	<0.001
Former	18,895 (21.3)	8,653 (19.5)	10,242 (23.0)	
Current	18,290 (20.5)	10,641 (23.9)	7,649 (17.2)	
Alcohol consumption				<0.001
None	55,467 (62.4)	28,112 (63.2)	27,355 (61.5)	
Mild to moderate	26,459 (29.8)	12,114 (27.3)	14,345 (32.3)	
Heavy	6,980 (7.8)	4,227 (9.5)	2,753 (6.2)	
Regular exercise	19,012 (21.4)	8,610 (19.4)	10,402 (23.4)	<0.001
BMI, kg/m^2^	23.2 ± 3.2	22.4 ± 3.1	24.1 ± 3.0	<0.001
<18.5	5,029 (5.7)	4,006 (9.0)	1,023 (2.3)	<0.001
18.5–22.9	37,353 (42.0)	22,169 (49.9)	15,184 (34.2)	
23.0–24.9	21,703 (24.4)	9,553 (21.5)	12,150 (27.3)	
≥25.0	24,821 (27.9)	8,725 (19.6)	16,096 (36.2)	
CCI	2.2 ± 1.9	2.7 ± 2.0	1.6 ± 1.7	<0.001
0–2	56,138 (63.1)	23,118 (52.1)	33,020 (74.3)	<0.001
≥3	32,768 (36.9)	21,335 (47.9)	11,433 (25.7)	

Data are expressed as the mean ± SD or n (%).*Low income denotes the lowest income quantile and Medicaid.BMI, body mass index; CCI, Charlson Comorbidity Index.

### Risk of fracture in TB survivors compared to matched controls

**Figure 2:**
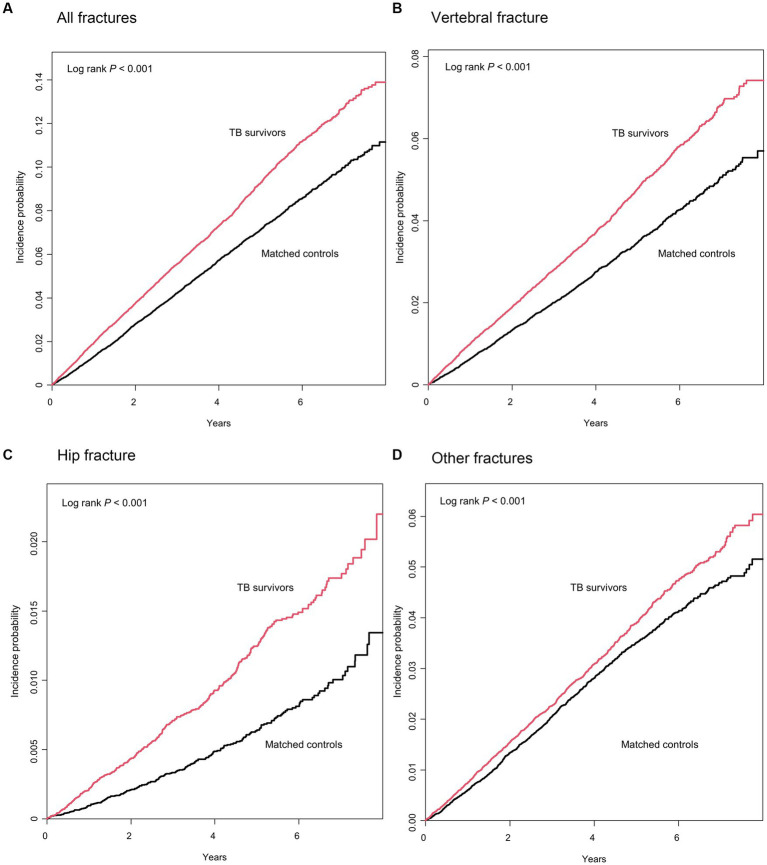
Cumulative incidence probability (%) of fracture in tuberculosis (TB) survivors versus matched controls. Year 0 indicates 1 year after TB diagnosis in TB survivors and 1 year after the time of being matched in matched controls, respectively.

The median duration of follow-up after 1 year of lag time was 3.3 (interquartile range, 1.5–5.2) years and 3.5 (interquartile range, 1.6–5.4) years for TB survivors and their matched controls, respectively. During the follow-up period, 6.6% (2,933/44,453) of TB survivors and 5.2% of the matched controls (2,329/44,453) experienced total fracture incidents, with an incidence rate of 19.3 and 14.6 per 1,000 person-years, respectively. Figure 2A shows a significant difference in the cumulative incidence probability of all fractures over 7 years between TB survivors and matched controls (log-rank *p* < 0.001). In line with this, TB survivors had a higher fracture risk [adjusted hazard ratio (aHR) 1.27, 95% confidence interval (CI) 1.20–1.34] compared to their matched controls (Table 2).

**Table 2:** Hazard ratios and 95% confidence intervals for the incidence of osteoporotic fractures in tuberculosis survivors compared to the matched control group.

	Participants	Fracture events	Follow-up duration (PY)	IR (/1,000 PY)	Model 1 (HR [95% CI])	Model 2 (HR [95% CI])
Total fracture						
Matched controls	44,453	2,329	159,004	14.6	1 (Reference)	1 (Reference)
TB survivors	44,453	2,933	151,851	19.3	1.32 (1.25–1.39)	1.27 (1.20–1.34)
Vertebral fracture						
Matched controls	44,453	1,144	161,910	7.1	1 (Reference)	1 (Reference)
TB survivors	44,453	1,519	155,322	9.8	1.39 (1.28–1.50)	1.35 (1.25–1.46)
Hip fracture						
Matched controls	44,453	215	164,281	1.3	1 (Reference)	1 (Reference)
TB survivors	44,453	386	158,104	2.4	1.87 (1.59–2.21)	1.65 (1.39–1.96)
Other fracture						
Matched controls	44,453	1,117	161,830	6.9	1 (Reference)	1 (Reference)
TB survivors	44,453	1,229	155,667	7.9	1.15 (1.06–1.24)	1.12 (1.03–1.21)

Data are presented as numbers or ratios, as appropriate.PY, person-years; IR, incidence rate per 1,000 person-years; HR, hazard ratio; CI, confidence interval; TB, tuberculosis. Model 1: crude model; Model 2: adjusted for age, sex, body mass index, smoking, alcohol consumption, socioeconomic status (income level and place of residence), and physical activity.

When categorizing the fractures into three groups by location—hip, vertebrae, and other fractures, TB survivors had a higher risk of vertebral fracture (aHR 1.35, 95% CI 1.25–1.46), hip fracture (aHR 1.65, 95% CI 1.39–1.96), and other fractures (aHR 1.12, 95% CI 1.03–1.21) (Table 2 and Figures 2B–D).

### Risk of fracture by stratified analysis

**Figure 3:**
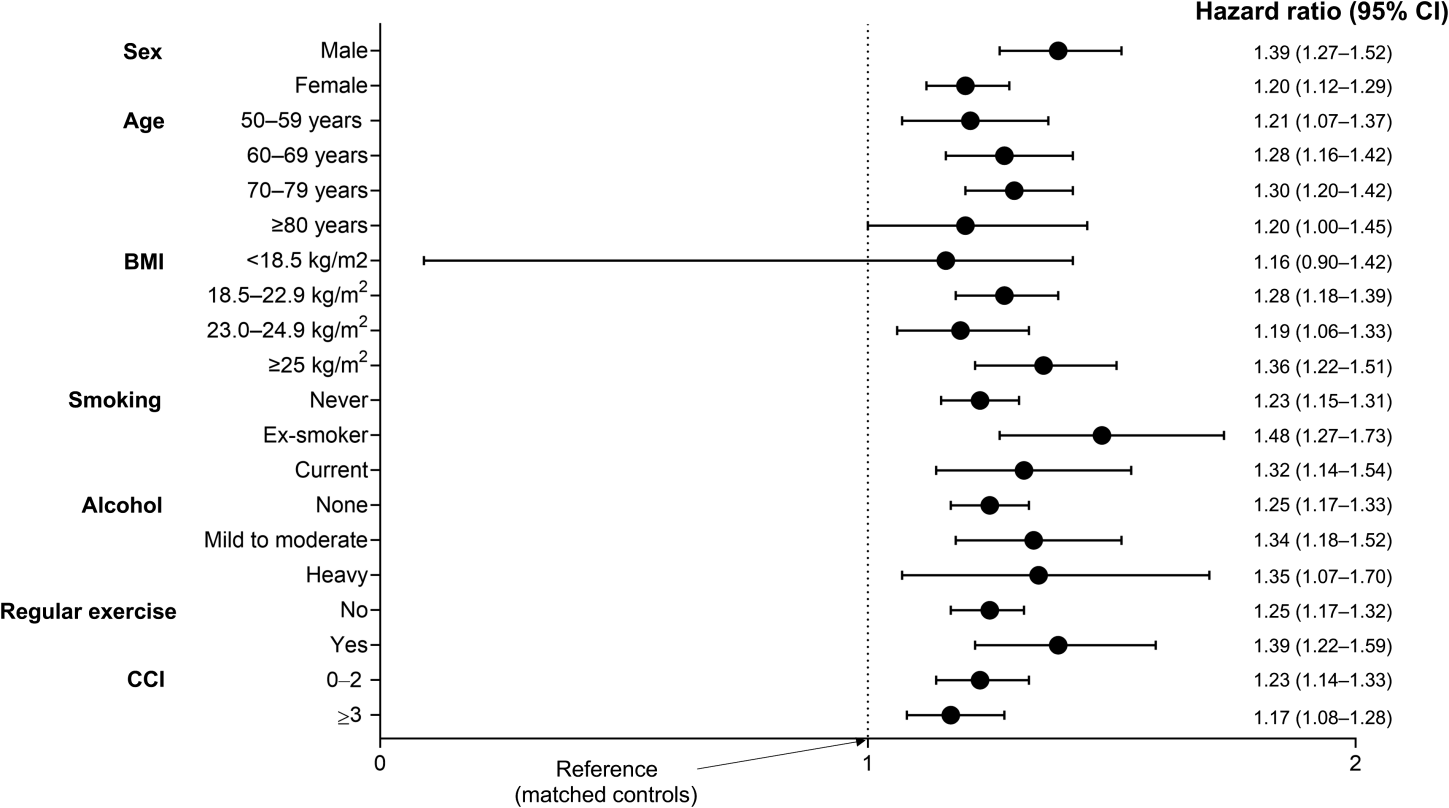
Subgroup analysis for the incidence of fracture in TB survivors compared to matched controls. Adjusted for age, sex, income level, residential area, smoking, alcohol consumption, regular physical activity, body mass index, and Charlson Comorbidity Index. CCI, Charlson Comorbidity Index; CI, confidence interval.

Figure 3 shows the various stratified analyses regarding the risk of fracture in TB survivors and their matched controls after adjusting for potential confounding variables in all subgroups. TB survivors exhibited a higher risk of fractures across subgroups.

### Fracture prediction model

Table 3 depicts factors associated with all fractures in TB survivors; factors associated with increased risk of all fractures were pulmonary TB vs. extrapulmonary TB (aHR 1.15, 95% CI 1.02–1.29), female vs. male sex (aHR 2.08, 95% CI 1.87–2.31), older age (aHR 2.05, 95% CI 1.86–2.26 for ages 60–74 years; aHR 3.68, 95% CI 3.29–4.12 for ages ≥75 years vs. ages 50–59 years), heavy alcohol consumption vs. none (aHR 1.26, 95% CI 1.08–1.48), lower income level (aHR 1.13, 95% CI 1.03–1.24 for income level Q1 [lowest] and aHR 1.11, 95% CI 1.01–1.22 for income level Q3 vs. income level Q4 [highest]), and CCI ≥ 2 vs. CCI = 0 (aHR 1.40, 95% CI 1.22–1.61). By contrast, regular physical activity was associated with a reduced risk of all fractures (aHR 0.89, 95% CI 0.80–0.98).

**Table 3:** Hazard ratios and 95% confidence intervals for the incidence of osteoporotic fractures in TB survivors.

		Participants	Fracture events	Follow-up duration (PY)	IR (/1,000 PY)	Univariable analysis		Multivariable analysis	
						HR (95% CI)	*P*	HR (95% CI)	*P*
Type of TB	Pulmonary TB	38,889	2,595	13,1725	19.7	1.17 (1.05–1.31)	<0.01	1.15 (1.02–1.29)	0.02
	Extra-pulmonary TB	5,564	338	20,126	16.8	1 (Reference)		1 (Reference)	
Sex	Male	27,016	1,202	90,001	13.4	1 (Reference)	<0.01	1 (Reference)	<0.01
	Female	17,437	1,731	61,850	28.0	2.01 (1.95–2.26)		2.08 (1.87–2.31)	
Age, years	50–59	15,947	581	58,586	9.9	1 (Reference)	<0.01	1 (Reference)	<0.01
	60–74	21,036	1,504	72,160	20.8	2.11 (1.91–2.32)		2.05 (1.86–2.26)	
	≥75 years	7,470	848	21,106	40.2	4.09 (3.68–4.54)		3.68 (3.29–4.12)	
BMI, kg/m^2^	<18.5	4,006	293	13,119	22.3	1.15 (1.02–1.31)	<0.01	1.13 (0.997–1.28)	<0.01
	18.5–22.9	22,169	1,472	76,033	19.4	1 (Reference)		1 (Reference)	
	23–24.9	9,553	561	32,939	17.0	0.88 (0.80–0.97)		0.86 (0.78–0.95)	
	≥ 25.0	8,725	607	29,761	20.4	1.05 (0.96–1.16)		0.93 (0.85–1.03)	
Smoking status	Never	25,159	2,090	87,562	23.9	1 (Reference)	<0.01	1 (Reference)	0.365
	Former	8,653	373	28,353	13.2	0.55 (0.49–0.62)		0.96 (0.84–1.10)	
	Current	10,641	470	35,936	13.1	0.55(0.50–0.61)		1.06 (0.93–1.21)	
Alcohol consumption	No	28,112	2,196	96,653	22.7	1 (Reference)	<0.01	1 (Reference)	<0.01
	Mild	12,114	526	40,874	12.9	0.57 (0.52–0.62)		0.97 (0.87–1.08)	
	Heavy	4,227	211	14,324	14.7	0.65 (0.56–0.75)		1.26 (1.08–1.48)	
Regular physical activity	No	35,843	2,474	122,592	20.2	1 (Reference)	<0.01	1 (Reference)	0.02
	Yes	8,610	459	29,260	15.7	0.78 (0.70–0.86)		0.89 (0.80–0.98)	
Income level	Q1 (lowest)	10,477	710	35,579	20.0	1.01(0.92–1.11)	0.144	1.13 (1.03–1.24)	0.06
	Q2	82,92	488	27,708	17.6	0.89 (0.80–0.99)		1.07 (0.96–1.20)	
	Q3	10,556	702	36,305	19.3	0.98 (0.89,1.08)		1.11 (1.01–1.22)	
	Q4 (highest)	15,128	1,033	52,260	19.8	1 (Reference)		1 (Reference)	
Residential area	Urban	19,223	1,136	66,806	17.0	1 (Reference)	<0.01	1 (Reference)	0.05
	Rural	25,230	1,797	85,045	21.1	1.24 (1.15–1.34)		1.077(0.999,1.161)	
CCI	0	5,304	231	18,764	12.3	1 (Reference)	<0.01	1 (Reference)	)<0.01
	1	8,734	462	30,390	15.2	1.23 (1.05–1.45)		1.07 (0.92–1.26)	
	≥2	30,415	2,240	10,2,697	21.8	1.77 (1.55–2.03)		1.40 (1.22–1.61)	

Data are presented as numbers or ratios, as appropriate.TB, tuberculosis; PY, person-years; IR, incidence rate per 1,000 person-years; HR, hazard ratio; CI, confidence interval; BMI, body mass index; CCI, Charlson Comorbidity Index. Multivariable analysis was adjusted for type of TB, age, sex, body mass index, smoking, alcohol consumption, socioeconomic status (income level and residential area), regular physical activity, and CCI.

**Figure 4:**
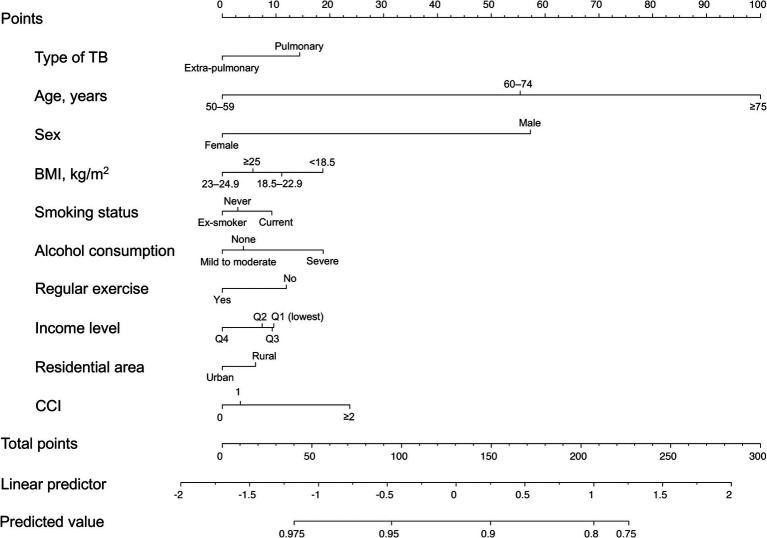
Nomogram for the prediction model of fracture probability. TB, tuberculosis; BMI, body mass index; CCI, Charlson Comorbidity Index.

The nomogram for the fracture risk in TB survivors is shown in Figure 4. The fracture risk prediction model showed good discrimination (C-statistic = 0.678; 95% CI = 0.668–0.689).

## Discussion

To our knowledge, this study is the first to evaluate fracture risk in TB survivors while comprehensively adjusting for potential confounders, including nutritional status, lifestyle habits, and comorbidities, and to develop an individualized risk prediction model for fractures in TB survivors. The fracture risk was significantly higher in TB survivors than in matched controls at all locations. Furthermore, a risk-prediction model for fractures based on related factors was reliable among TB survivors.

The pathogenesis of fractures in TB survivors is not fully understood, but three potential mechanisms are as follows. The first possible mechanism is chronic inflammatory reaction induced by TB infection. Studies have suggested that pro-inflammatory markers such as IL-6, IL-1, and TNF-alpha act as modulators of osteoblasts and osteoclasts, leading to up-regulation of osteoclast activation and osteoporosis (9, 39). It has been suggested that TB infection chronically elevates systemic inflammatory cytokines resulting from non-resolving inflammation as an outcome of intracellular survival strategies (3, 40). Remarkably, these inflammatory reactions persist even after the completion of anti-TB treatment, with chronic inflammation acting as an osteoclastic stimulator in TB survivors, resulting in increased fracture risk (39, 41).

The second presumable mechanism is weight loss caused by TB infection. TB survivors suffer from anorexia and weight loss and remain underweight compared with healthy controls (42, 43). In addition, TB survivors have a higher prevalence of sarcopenia than those without TB infection (44). Several studies have shown that low body weight and BMI increase osteoporosis and fracture risks (11), so the low BMI of TB survivors could contribute to an elevated fracture incidence in this population.

The third potential mechanism is Vitamin D deficiency. In previous studies, low serum vitamin D levels (≤ 25 nmol/L) exhibited a significant association with an increased risk of TB infection. Even after completing TB treatment, survivors demonstrate a higher incidence of vitamin D deficiency than the general population (45–47). Vitamin D deficiency in TB survivors can alter calcium absorption, causing a negative calcium balance and bone reabsorption, leading to increased osteoporosis and fracture risks (48).

In our study, the hazard of hip fractures in TB survivors relative to matched controls was higher than that of vertebral and other fractures. The mechanism is not fully understood; however, it is suspected that TB survivors undergo increased pro-inflammatory cytokine and osteoclastic cell activities, resulting in net resorption and a decrease in the endocortical surface by 25–40% at the femoral neck and distal radius (49). In addition, altered calcium absorption due to vitamin D deficiency may lead to decreased bone reabsorption in the cortical bone, weakening the cortical bone of the hip more frequently than the vertebral bones (48).

A highlight of our study is that we speculated on the risk factors for fractures in TB survivors and developed an individualized estimation prediction model. Although there is an increased fracture risk in TB survivors compared with the general population, no studies have attempted to develop risk estimation models for TB survivors. The major advantage of our study is that, to the best of our knowledge, we have developed a fracture risk estimation model based on clinical variables that can be easily obtained from real-world clinics. Accordingly, our model can be applied to TB survivors in real-world clinics.

Our study had some limitations. First, our follow-up period (median 3.4 years after a 1-year lag period) was relatively short, considering it takes a long time to develop osteoporosis. Second, information on vitamin D levels was unavailable due to the use of claims data, limiting the ability to conclude a direct correlation. Third, this study could not include all possible confounding factors, such as previous falls and physical function status, because of the use of claims data. In addition, the causes of the fractures were incomplete, and fractures resulting from other causes, such as traffic accidents or other sources of trauma, were not excluded. However, fractures caused by traffic accidents or violence were not covered by the NHIS and were not included in this study. Despite these limitations, our study has several strengths, including the use of a large and representative database of the entire Korean population, near-complete follow-up, and adjustments for lifestyle variables, including BMI, smoking, alcohol consumption, and regular physical activities.

This study clearly demonstrated TB survivors are a high-risk group for fractures, so they would benefit from following clinicians’ attempts to reduce fracture risk. First, effective and complete TB treatment is necessary to reduce the inflammatory cytokines induced by TB infection. Second, physicians need to pay attention to weight gain in TB survivors and provide proper nutritional advice during and even after TB treatment. Third, considering TB survivors’ high risk of vitamin D deficiency, constructive suggestions for sustained bone mineral density in postmenopausal women or those aged over 50 years for TB survivors are necessary. Future studies are warranted to investigate how such strategies can be incorporated into our daily practice to monitor and prevent osteoporotic fractures in TB survivors effectively.

In conclusion, this nationwide population-based cohort study demonstrated that TB survivors have a higher fracture risk than the general population. Future studies are necessary to understand better the precise mechanisms underlying the association between TB and fractures.

## Data availability statement

The raw data supporting the conclusions of this article will be made available by the authors, without undue reservation.

## Ethics statement

The studies involving humans were approved by the Institutional Review Board of the Hanyang University Hospital (application no. HYUH-2019-10-047). The studies were conducted in accordance with the local legislation and institutional requirements. The ethics committee/institutional review board waived the requirement of written informed consent for participation from the participants or the participants’ legal guardians/next of kin because The review board waived the requirement for written informed consent because the data were publicly available and anonymized under confidentiality guidelines.

## Author contributions

HC: Data curation, Formal analysis, Funding acquisition, Visualization, Writing – original draft, Writing – review & editing. JS: Conceptualization, Formal analysis, Methodology, Visualization, Writing – original draft, Writing – review & editing. J-HJ: Data curation, Formal analysis, Resources, Writing – original draft, Writing – review & editing. KH: Conceptualization, Data curation, Formal analysis, Investigation, Software, Writing – original draft, Writing – review & editing. WC: Data curation, Formal analysis, Methodology, Resources, Writing – original draft, Writing – review & editing. HRL: Investigation, Software, Writing – original draft, Writing – review & editing. JY: Investigation, Project administration, Resources, Software, Writing – original draft, Writing – review & editing. YY: Data curation, Formal analysis, Investigation, Resources, Software, Validation, Writing – original draft, Writing – review & editing. HL: Conceptualization, Data curation, Formal analysis, Funding acquisition, Investigation, Methodology, Project administration, Resources, Software, Writing – original draft, Writing – review & editing. DS: Conceptualization, Funding acquisition, Investigation, Methodology, Project administration, Resources, Software, Supervision, Validation, Visualization, Writing – original draft, Writing – review & editing.
